# Development of an Apparatus for Crop-Growth Monitoring and Diagnosis

**DOI:** 10.3390/s18093129

**Published:** 2018-09-17

**Authors:** Jun Ni, Jingchao Zhang, Rusong Wu, Fangrong Pang, Yan Zhu

**Affiliations:** 1 College of Agriculture, Nanjing Agriculture University, Nanjing 210095, China; nijun@njau.edu.cn (J.N.); wrs375207731@163.com (R.W.); pangfangrong@njau.edu.cn (F.P.); 2 National Engineering and Technology Center for Information Agriculture, Nanjing 210095, China; 3 Jiangsu Key Laboratory for Information Agriculture, Nanjing 210095, China; 4 Jiangsu Collaborative Innovation Center for the Technology and Application of Internet of Things, Nanjing 210095, China; 5 Nanjing Institute of Agricultural Mechanization of National Ministry of Agriculture, Nanjing 210014, China; zhangjc9@163.com

**Keywords:** crop growth index, canopy reflectance spectra, multispectral sensor, apparatus for monitoring and diagnosis, field experiment

## Abstract

To non-destructively acquire leaf nitrogen content (LNC), leaf nitrogen accumulation (LNA), leaf area index (LAI), and leaf dry weight (LDW) data at high speed and low cost, a portable apparatus for crop-growth monitoring and diagnosis (CGMD) was developed according to the spectral monitoring mechanisms of crop growth. According to the canopy characteristics of crops and actual requirements of field operation environments, splitting light beams by using an optical filter and proper structural parameters were determined for the sensors. Meanwhile, an integral-type weak optoelectronic signal processing circuit was designed, which changed the gain of the system and guaranteed the high resolution of the apparatus by automatically adjusting the integration period based on the irradiance received from ambient light. In addition, a coupling processor system for a sensor information and growth model based on the microcontroller chip was developed. Field experiments showed that normalised vegetation index (NDVI) measured separately through the CGMD apparatus and the ASD spectrometer showed a good linear correlation. For measurements of canopy reflectance spectra of rice and wheat, their linear determination coefficients (*R*^2^) were 0.95 and 0.92, respectively while the root mean square errors (RMSEs) were 0.02 and 0.03, respectively. NDVI value measured by using the CGMD apparatus and growth indices of rice and wheat exhibited a linear relationship. For the monitoring models for LNC, LNA, LAI, and LDW of rice based on linear fitting of NDVI, *R*^2^ were 0.64, 0.67, 0.63 and 0.70, and RMSEs were 0.31, 2.29, 1.15 and 0.05, respectively. In addition, *R*^2^ of the models for monitoring LNC, LNA, LAI, and LDW of wheat on the basis of linear fitting of NDVI were 0.82, 0.71, 0.72 and 0.70, and RMSEs were 0.26, 2.30, 1.43, and 0.05, respectively.

## 1. Introduction

The basic requirement of precision agriculture is to rapidly acquire accurate and reliable crop-growth information in a convenient way at low cost; this is also key to realising accurate management and regulation of crops [1,2,3]. For a long time, crop-growth information was acquired through destructive sampling in the field and indoor biochemical measurements. Although the results are reliable, these methods are laborious, take a long time and exhibit an undesirable lack of timeliness. In recent years, non-destructive monitoring technologies based on feature identification through reflectance spectra have shown various advantages, including non-destructibility, convenient access to information, and good real-time performance. Thus, they have been widely used in research into the monitoring of crop-growth indices and estimation of agricultural mechanisms [4,5,6,7,8]. Such research is carried out mainly based on existing ground-based object spectrometers with various advantages such as abundant wavebands, high resolution, and measurement accuracy [9,10,11]. However, spectrometers also exhibit a few disadvantages, including high price, complex structure, tedious operation, and inconvenient field application. Development of spectral monitoring for crop growth promotes the development and application of some spectrometers for crop-growth monitoring. For example, the multispectral radiometer produced by Cropscan (Rochester, MN, USA) in the United States can obtain canopy spectral reflectance in 16 wave bands [12]. Staff at NASA’s Goddard Space Flight Center (Greenbelt, MD, USA), have developed a two-band laser sensor for measuring vegetation indices. The system uses a semiconductor laser, with characteristic wavelengths of 660 nm and 780 nm [13]. The Finnish Geospatial Research Institute (Helsinki, Finland) designed a two-band laser radar powered by a supercontinuum source, working at wavebands of 600 nm and 800 nm, to distinguish vegetation and inorganic materials by constructing the NDVI [14]. Decagon Devices (Pullman, WA, USA) have developed a portable SRS radiometer, operating at two wavebands (630 nm and 800 nm), which calculates NDVI by measuring canopy radiation intensity [15]. By using the two-band spectrometer–Greenseeker produced by Trimble Navigation (Sunnyvale, CA, USA) in the United States, the normalised vegetation index (NDVI) is constructed through measuring canopy reflectance of crops [16,17,18]. The N-Sensor spectrometer produced by Yara (Haninghof, Germany) utilising sunlight as its light source is composed of five detectors, one of which is used for detecting solar incident light and another four to measure radiation reflected from plant canopies. The spectrometer works on 20 different wave lengths in the range of 450 to 900 nm [19]. Konic Minolta (Osaka, Japan) developed a spectrometer (SPAD-502) with an LED light source to measure spectral absorptivity of leaf chlorophyll at wave bands of 650 and 940 nm [20]. The Crop Circle ACS-470 spectrometer produced by Holland Scientific (Lincoln, NE, USA) is used for measuring canopy reflectance spectra of crops [21,22]. The CropSpec based on a laser-modulated light source was developed by TOPCON (Tokyo, Japan) [23]. The dual-band crop canopy analyzer is developed by China Agricultural University (Beijing, China) [24,25]. The development and application of these spectrometers provide powerful technological support for precision management and information acquisition of crops [26,27,28,29]. However, these items of equipment only display single functions and output canopy reflectance or spectral vegetation indices. Therefore, they are generally applied in scientific research and cannot be directly applied to agricultural production due to the lack of crop-growth information. In addition, the high cost of such equipment fails to meet the requirements of precision agriculture for acquiring field information at low cost.

Based on the monitoring mechanism used on reflectance spectra for crop-growth information, apparatus for crop-growth monitoring and diagnosis (CGMD) was developed by using findings of the research group relating to spectral bands sensitive to rice and wheat growth indices. By applying the apparatus, various growth indices including NDVI, leaf nitrogen content (LNC), leaf nitrogen accumulation (LNA), leaf area index (LAI), and leaf dry weight (LDW) of rice and wheat can be non-destructively acquired in real-time. Then, nitrogenous fertiliser doses can be calculated based thereon. The apparatus exhibits a simple structure, high integration, and good cost performance, and therefore is conveniently deployed on field operations. In addition, it has also been popularised in agricultural production estimation.

## 2. Measurement Principle of the CGMD Apparatus

**Figure 1:**
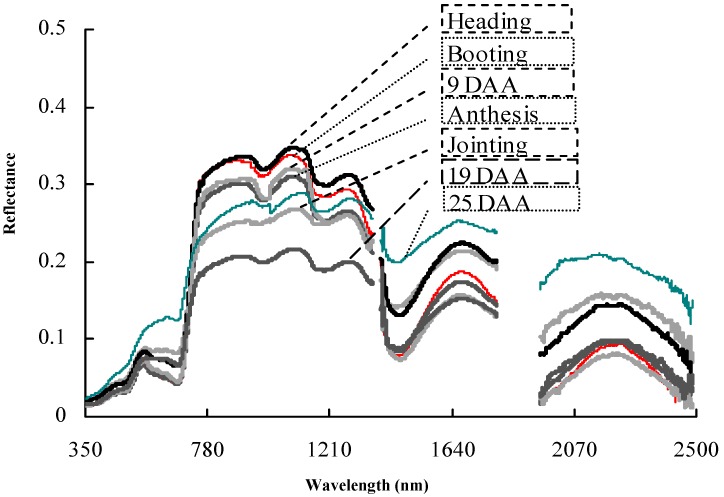
Dynamic changes of canopy spectral reflectance of wheat variety Ningmai 9 at different growth stages. Note: (1) DAA represents days after anthesis (2) This result was obtained under conditions applying 90 kg·ha^−1^ of pure nitrogen content fertiliser with a dressing ratio of 1:1 (3) measurements were carried out at 12:00 in the growth stage on sunny days without wind and cloud.

During crop growth, the changes of various indices including LNC, LNA, LAI, and LDW probably cause changes in the reflectance spectra of crops [30]. Figure 1 displays canopy spectral reflectance curves of wheat variety Ningmai 9 from its elongation stage to the late filling stage as measured by the FieldSpec Pro FR2500 back-hanging field hyperspectral radiometer (ASD spectrometer) produced by ASD Inc., (Alexandria, VA, USA). It can be seen from the figure that canopy reflectance spectra showed significant disparities with the changes of crop-growth information in different growth stages. According to existing research [31,32,33], spectral reflectance at wave bands of 720 and 810 nm has a close relationship with the growth information of rice and wheat. In addition, there is a specific relationship between dynamic changes of spectral reflectance and changes in the growth information. Therefore, by using photovoltaic conversion devices, information about reflectance at the above wave bands can be acquired and then used to infer, by inversion, the growth condition of rice and wheat.

In general, reflectivity refers to the ratio of reflected energy of objects to incident energy while spectral reflectance refers to that measured in a certain wavelength interval [34]. Lambertian reflection occurs after the solar spectrum with wavelength *r* is projected parallel to the canopy leaves of a crop. According to the Lambert cosine law, the following formula can be obtained:

(1) $$\frac{d\varphi_{r}}{dAd\omega }=L_{r}\times \cos\theta$$

where, $L_{r}$, $\varphi_{r}$ and A separately represent reflected radiance of canopies, optical power, and light projected area, while ω and θ refer to solid angle and reflection angle, respectively.

$M_{r}$ is defined as the total reflected optical power from a unit area of the crop canopy to an idealised upper hemispherical space. Therefore, the following formula can be obtained:

(2) $$M_{r}=\iint \frac{d\varphi_{r}}{dA}=\iint L\cos\theta d\omega =L\int_{0}^{0.5\pi }\cos\theta \sin\theta d\theta \int_{0}^{2\pi }d\phi_{r}=\pi L_{r}$$

The optical power of solar spectrum with wavelength λ which is projected parallel to a unit area of the canopy is represented as $E_{r}$, so reflectivity $\rho_{r}$ of crop canopies for solar spectrum with wavelength λ can be defined, according to the definition of reflectivity, as follows:

(3) $$\rho_{r}=\frac{\pi L_{r}}{E_{r}}$$

Formula (3) shows that canopy reflectivity $\rho_{r}$ can be calculated after acquiring the irradiance of sunlight projected onto crop canopies and the radiance of canopy surfaces using the sensors.

## 3. Design of the CGMD Apparatus

### 3.1. Overall Design

**Figure 2:**
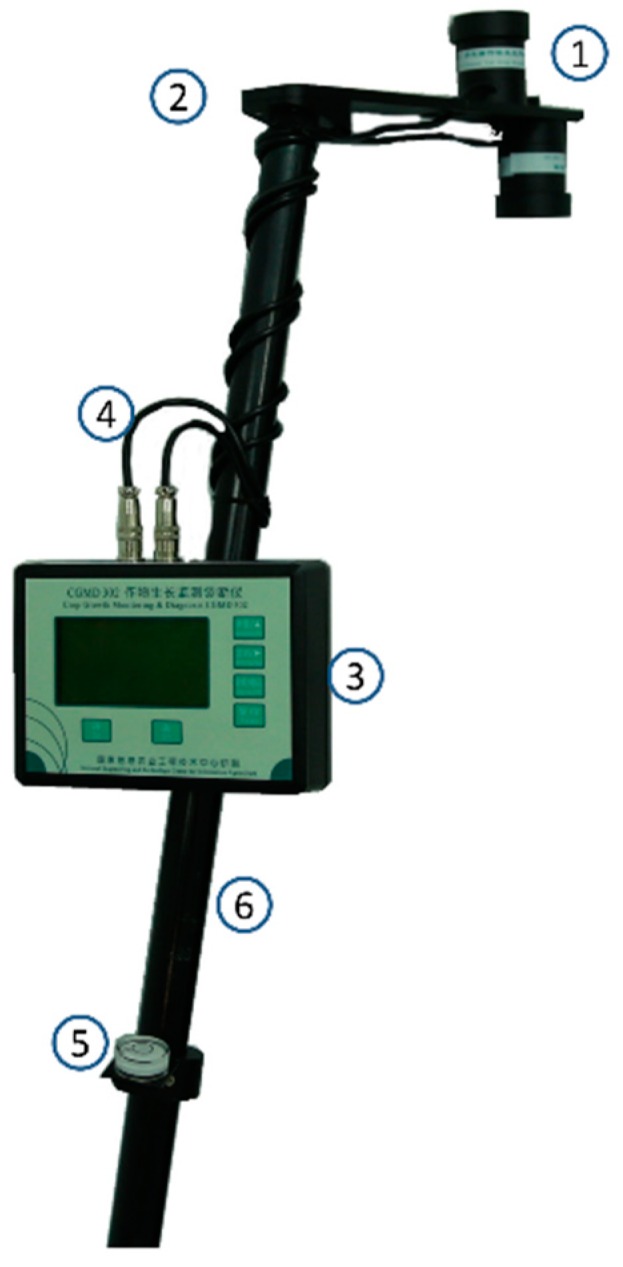
External view of the CGMD apparatus: ➀ Multispectral sensor; ➁ Sensor support; ➂ Processor system; ➃ Shielded cable; ➄ Gradient measurement device; ➅ Support bar.

The CGMD apparatus is composed of a multispectral sensor, a signal processing circuit, a processor system, a sensor support, a gradient measurement device, and a support bar, as shown in Figure 2.

### 3.2. Multispectral Sensor

**Figure 3:**
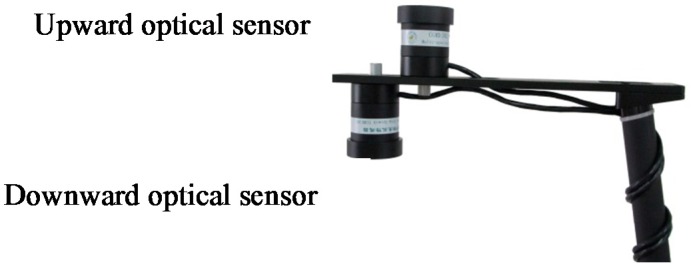
Multispectral sensors.

Using sunlight as the light source, the multispectral sensor comprises of an upward and a downward optical sensor: the former receives radiation information about the sunlight at 720 and 810 nm while the latter receives radiation information reflected from the crop canopy at corresponding wave bands. The radiation information is processed after being converted to electrical signals through the photoelectric detector, to obtain the characteristic spectral reflectance of the crop canopies. Based on canopy reflectance and the coupling crop-growth model, growth information can be acquired including LNC, LNA, LAI, and LDW. The sensors are integrated to make it convenient for integration and transplantation and suitable for a field testing environment, as shown in Figure 3.

#### 3.2.1. Downward Optical Sensor

**Figure 4:**
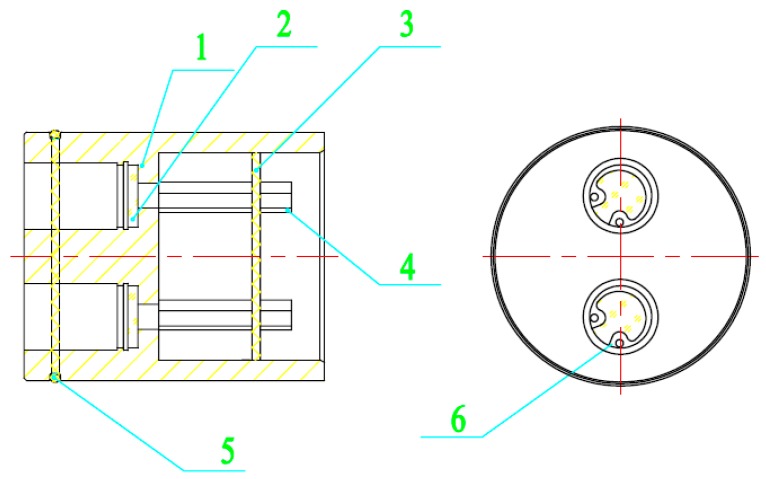
Structure of the downward optical sensor. 1: shield casing; 2: spectral filter; 3: the base of the photoelectric detector; 4: set screws; 5: filter; 6 compression spring.

The downward optical sensor is vertical to target the canopy so as to measure the reflectance spectra of the crop canopies. In addition, the downward optical sensor is composed of two detector lenses for detection at characteristic wavelengths of 720 and 810 nm, respectively. The signal-to-noise ratio (SNR) of any input optical radiation information is improved by using an optical filtering technique. In addition, each detector lens consists of only a spectral filter and photoelectric detectors and thereby exhibits a simple light path. This structure guarantees the reliability of signal transmission and overcomes the drawbacks of existing crop-growth spectral sensors including complex light paths and usage of numerous optical devices, as shown in Figure 4. A narrow-band light filter made from optical glass is chosen as the spectral filter. The central wavelength, transmittance at the central wavelength, peak transmittance, bandwidth, and cut-off rate of the spectral filter are characteristic wavelength ±2 nm, 65% to 70%, 65% to 70%, 9 nm, and lower than 0.00001%, respectively. The application of the spectral filter not only improves the measurement precision of the sensor by greatly inhibiting the entrance of spectral information at other wavebands in the detector lens but also guarantees uniform sensitivity of the sensor. As for the photoelectric detectors, the photodiode type is selected whose spectral response range is from 400 nm to 1100 nm while its sensitivity and maximum short-circuit current are 0.55 A/W and 120 μA, respectively. Two photoelectric detectors are installed on the same base, which improves the reliability of the system. Determination of aperture parameters is the key to the design of the detector lens, which is necessary to guarantee, not only the high resolution of the sensor system, but also the signal strength of the sensor.

**Figure 5:**
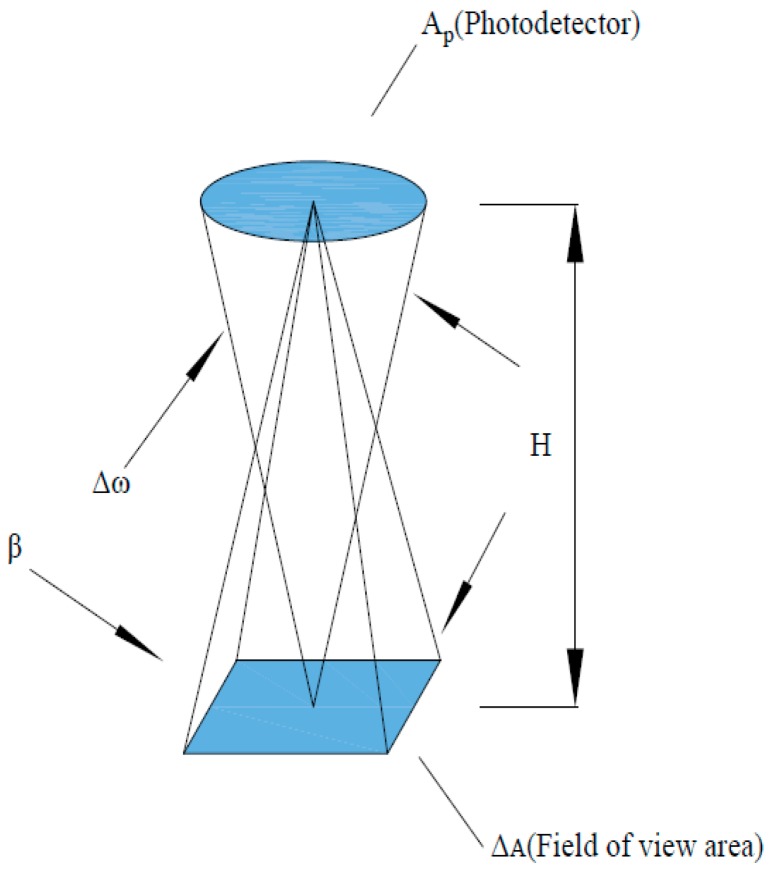
Transmission light path of the reflection-type photoelectrical detecting system.

The downward optical sensor receives reflected information from crop canopies when sunlight is projected onto the crop canopies. It can be seen from the transmission path of the reflection-type photoelectrical detecting system in Figure 5 that relationships among the diameter, field angle and the field of view area of the photoelectric detectors are shown as follows [35]:

(4) $$\Delta w=\frac{A_{p}}{H^{2}}$$

(5) $$\beta =\frac{\Delta A}{H^{2}}$$

where, ∆*w*, *Ap*, *β*, ∆*A*, and *H* separately represent the total energy received by the photoelectric detectors, the effective geometric area of the sensor, field angle, field of view area of the sensor, and the vertical distance from the sensor to canopies, respectively.

**Figure 6:**
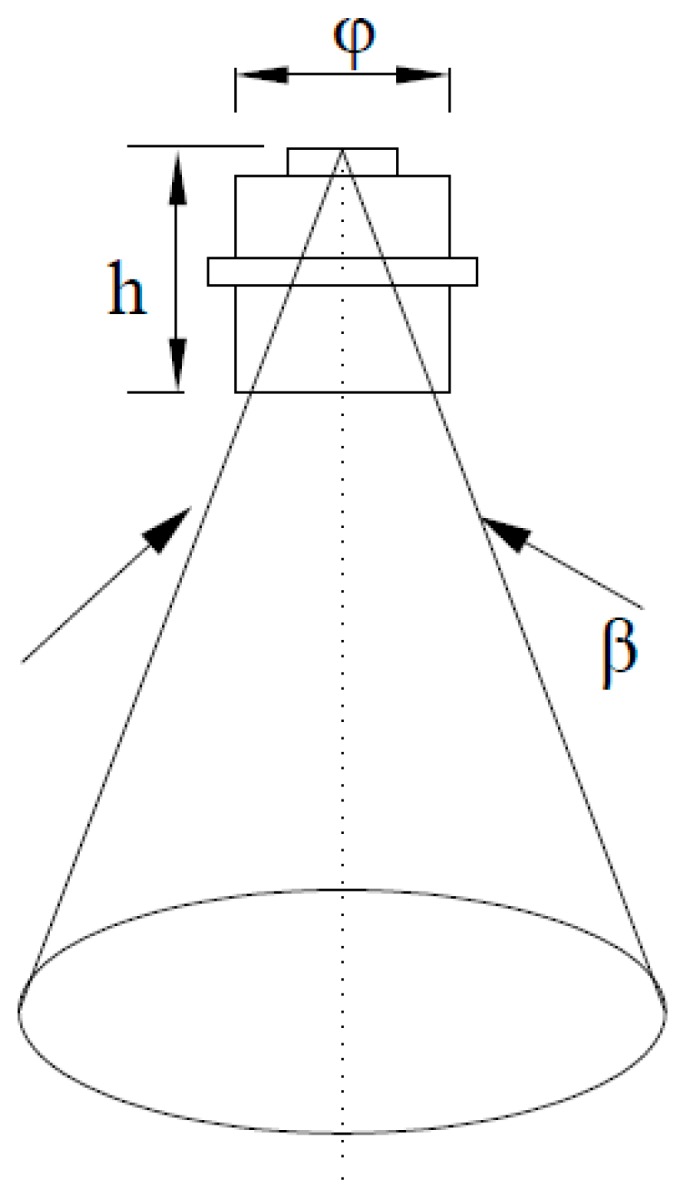
Parameter estimation of detection lenses.

The total energy *E* received by the photoelectric detectors is directly proportional to ∆*w*·∆*A*: that is, *E* ∝ ∆*w*·∆*A* = *A_p_β*. For the selected photoelectric detectors, the larger the designed field angle *β*, the stronger the spectral information obtained when the effective geometric area is unchanged, which is conducive to the acquisition and processing of signals. However, field crops show different growth states, so disparities of objects are ignored in large detection field areas and the acquired information tends to be inaccurate. To guarantee sensitivity and identification effect of detection, the field angle of the sensor is designed as 27° by comprehensively considering the performances from these two perspectives. When the sensor is placed 1 to 1.2 m above the crop canopy, the field of view covers a circular region whose diameter is about half of the height. Parameters of the detection lenses are shown, as estimates, in Figure 6.

When the downward optical sensor is 1 m above the crop canopy, the diameter of the detection area is 0.5 m. If aperture diameter and hole depth are set as ϕ and *h*, respectively, the following formulae can be obtained:

(6) $$\frac{250}{1000+h}=\tan\frac{\beta }{2}$$

(7) $$\frac{\varphi /2}{h}=\tan\frac{\beta }{2}$$

It can be seen from Equations (6) and (7) that the aperture diameter and hole depth are 12.8 m and 26 mm, respectively. The structure guarantees that the sensor has a high resolution and strengthens the signal from the sensor.

#### 3.2.2. Upward Optical Sensor

**Figure 7:**
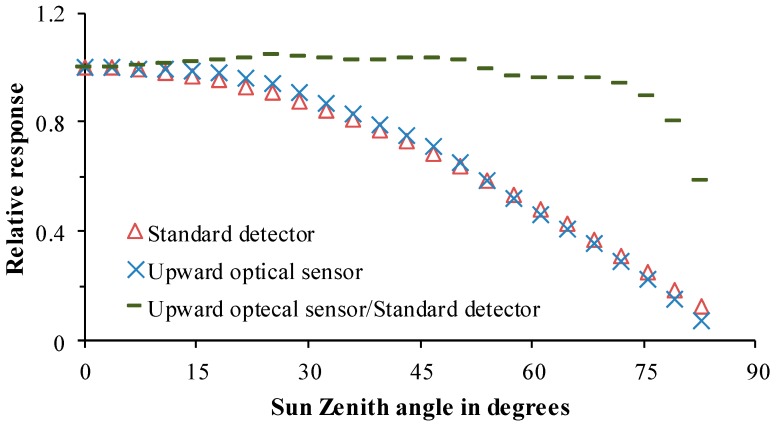
Cosine performance of the upward optical sensor.

The upward optical sensor is composed of two detection lenses for detection at characteristic wavelengths of 720 and 810 nm, respectively. To eliminate the influence of structures and textures of the sensor on optical signal transmission, structural parameters and component matching for detection lenses of the sensor are the same as those of the downward optical sensor. The upward optical sensor collects radiation information from the incident sunlight. To reduce the influence of changing solar elevation angle on incident light information, a tabulated cosine corrector is designed to correct the spectra. The transmittance and visual angle of the cosine corrector (made of polytetrafluoroethylene) are 75% and 180°, respectively. It is pasted on the aperture surface of the detection lenses. The better the cosine properties of the upward optical sensor, the higher the correlation between output light current and light intensity shot onto the sensor surface. In ideal conditions, when the incidence zenith angle of sunlight changes in the range of 0 to 90°, the output of the sensor shows a cosine correlation with the incident zenith angle of the sunlight while it exhibits a relative error of zero against the calibration curve of the standard cosine corrector. It can be seen from cosine performance of the upward optical sensor in Figure 7 and Table 1 that when solar zenith angle is less than 70° (solar height larger than 20°), the root-mean-square error (RMSE) is lower than 0.03 and in that condition, cosine correction approaches an ideal state. While, when the solar zenith angle is larger than 70°, the changing trend of the curve deviates from its ideal state, so it is not suggested that measurement is undertaken in such conditions.

### 3.3. Signal Processing Circuit

As the narrow-waveband beam split device is applied in the multispectral crop-growth sensor, only a little energy is projected onto the sensitive elements after spectral information is transmitted through the optical structure of the sensor. After transmitting the energy through the photoelectric sensor, the incident light current output by the upward optical sensor is at the μA level while current signals of reflected light output by the downward optical sensor reach the nA level. Various factors exert a significant influence on the processing and test result analysis of weak signals including stray light from the environment, amplifier noise, thermal noise in the circuits, and high-frequency interference effects in digital circuits. Rice and wheat are planted in extensive areas across China. To carry out tests under different eco-conditions and in different time windows, the system needs to not only be strongly capable of resisting interference, but also have certain adaptive capacity to luminous environmental changes so as to ensure the universality of the instrument. Spectral reflectance of crop canopies is acquired through irradiance of sunlight acquired by dividing the solar irradiance collected using the upward optical sensors by the radiance of canopy surface obtained by the downward optical sensor. As division operator is sensitive to data changes in both numerator and denominator, there is an onus on the stability of the signal processing circuit. Therefore, the signal processing circuits for the crop-growth sensors should have various characteristics such as low circuit noise, strong resistance against interference, high detection precision, wide detection range, and good environmental suitability.

**Figure 8:**
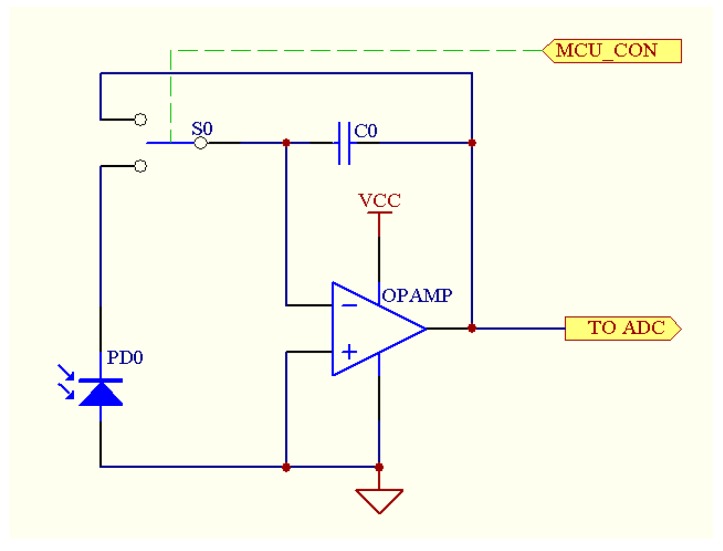
Integral-type signal processing circuit.

In the study, an integral-type signal processing circuit was designed to transmit the output signals from the multispectral sensor, as shown in Figure 8. According to changes of irradiance of the ambient light, the integration periods of the circuit can be automatically adjusted so as to change the gain of the system and accumulate photocurrent signals within an appropriate voltage range (⅓ to ⅔ of AD range). Thus, it is guaranteed that the apparatus has a high resolution under ambient light with different irradiances. The integral-type signal processing circuit shows a low sampling rate and bandwidth. In addition, due to the special I-U structure, it offers both high precision and strong resistance to high-frequency noise and power frequency interference.

According to the sensitive wavebands of the multispectral sensor, the selected photodiode-PD0 shows a strong sensitivity within the range of 700 to 850 nm. In addition, the circuit is connected in photovoltaic mode and weak current signals output from the photodiode exhibit a strong linear correlation with ambient light intensity. As an amplifier an OPAMP is used as the load component of the photodiode, to reduce the influence on small light currents, an amplifier with a large input impedance and small input bias current was chosen. The AD8626 from ADI Inc. was used whose input stage was a junction field effect transistor with a small input bias current (<1 pA), input offset current (<0.5 pA), and offset voltage (<500 μV). An analog electronic switch (S0) is connected between the photodiode and the amplifier to control the time of capacitance charge-discharge so that light current is transmitted to voltage. The resistance effect of the switch means that, to reduce consumption of weak light current, an analog electronic switch with low on-resistance and cut-off leakage current was selected: an analog electronic switch (MAX4620, Maxim Integrated, San Jose, CA, USA) was selected here. The device has advantages including low on-resistance (<70 Ω), high flatness of channel resistance (0.5 Ω), and low leakage current (<10 pA). The analog electronic switch is controlled by a single-chip microcomputer programmed to adjust the integral time of the circuit, and the integral time is designed as multiples of 20 ms (the power frequency in China is 50 Hz) to decrease power frequency interference. In conditions where the initialised integral time of the circuit is set as 20 ms, the integral time increases to 40 ms when the output voltage is detected to be lower than ⅓ VREF (AD reference voltage). Next, the output voltage is redetected and the integral time is doubled again if the value is still lower than ⅓ VREF. In the measurement, the integral time reduces by half if the output voltage is larger than ⅔ VREF. By doing so, it guarantees that in different light environments, the CGMD apparatus always has a high resolution as it can adjust the integral time to ensure an output voltage within ⅓ to ⅔ VREF.

### 3.4. Processor System

The processor system is mainly designed to control the collection, processing, and storage of spectral information, the spectral coupling of crop-growth monitoring model, calculation using the nitrogen diagnosis model, display of measurement results, and function identification and switching of keys. The main control chip in the processor system is the single-chip microcomputer STC12C5A60S2 produced by STC, which enables the CGMD apparatus to have two operating modes: function switch and parameter presupposition, realised through three multiplexing keys: measurement/▲, monitoring/► and diagnosis/affirmation. The measurement/▲ converts the system to its measurement state, starts the monitoring sub-program, and displays the measured canopy reflectance and NDVI values on the liquid-crystal display (LCD). “Cycle + 1” is multiplexed under parameter presupposition mode to perform cyclic addition of 1 for the parameter values of selected parameter bits. By pressing the key monitoring/►, the system is converted to its monitoring state and this stops the measurement. Next, the data at the last moment in its measurement state, are coupled with the crop-growth spectral model to calculate and display LNC, LNA, LAI, and LDW values on the LCD. The function “rotate right” is conducted in parameter presupposition mode to cyclically shift the current cursor to the right to the next alternative parameter bit. The system is changed to a parameter presupposition state or diagnosis state via the key “diagnosis/affirmation”. In parameter presupposition state, parameters are input and diagnosed, while in the diagnosis state, input parameters and monitoring results are coupled with the diagnosis model to compute and display the dressing contents of nitrogenous fertiliser on the LCD.

The software running the processor system consists of five parts, including an initialiser, a main program, a key service program, an intelligent monitoring program, and a data processor. The initialiser is used for boot initialisation of the system, and monitoring and displaying information about environmental temperatures. The main program is used to control state machines in measurement, resting, monitoring, setting, and diagnosis states, and so on. The key service program is employed in various ways including interrupting response, reading and explaining keys, and as a status switch. The intelligent monitoring program is designed to automatically adjust the integration period based on the irradiance of the ambient light to control the gain in the system. In addition, the data processor is used in the pre-processing of spectral data, calculation of canopy reflectance, and the coupling of reflectance with the crop-growth spectral model and the diagnosis model.

**Figure 9:**
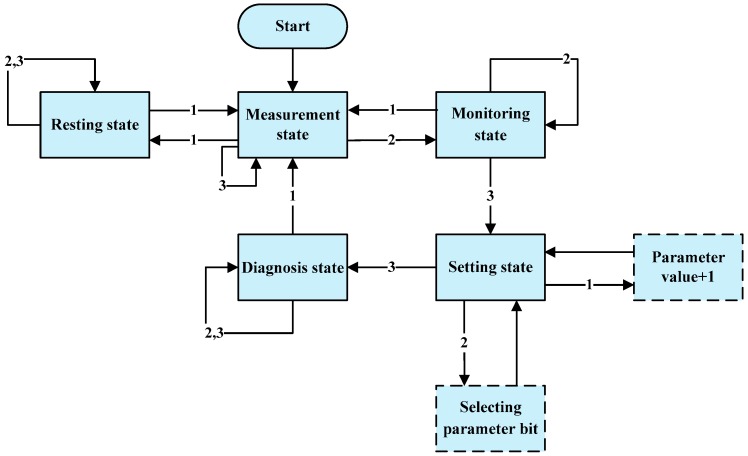
State machines of the processor system software.

A switch of state machines from the main program, and invoking of the intellectual monitoring program and the data processor are realised through keys, as shown in Figure 9: “1”, “2”, and “3” represent valid measurement/▲, monitoring/►, and diagnosis/affirmation, respectively. In the measurement state, by constantly running the intellectual monitoring program, canopy reflectance and NDVI values are displayed. In the monitoring state, agronomic parameters (LNC, LNA, LAI, and LDW) are output by coupling the above results with the agriculture model. In the setting state, by using the keys: “measurement/▲” and “monitoring/►”, the input parameters of the diagnosis model are set, while the dressing contents of nitrogenous fertiliser are displayed in the diagnostic state.

### 3.5. Calibration of the CGMD Apparatus

**Figure 10:**
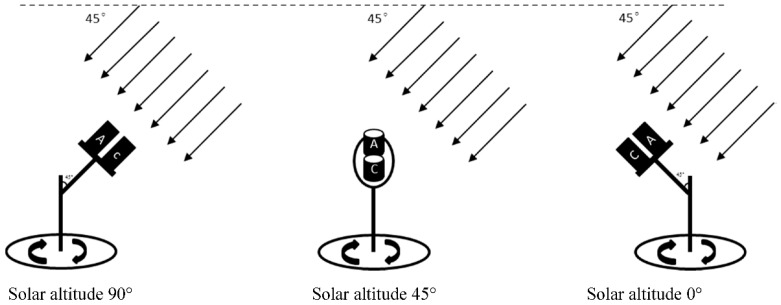
Calibration principle of the upward optical sensor. A and C represent the ASD spectrometer and the upward optical sensor, respectively.

**Figure 11:**
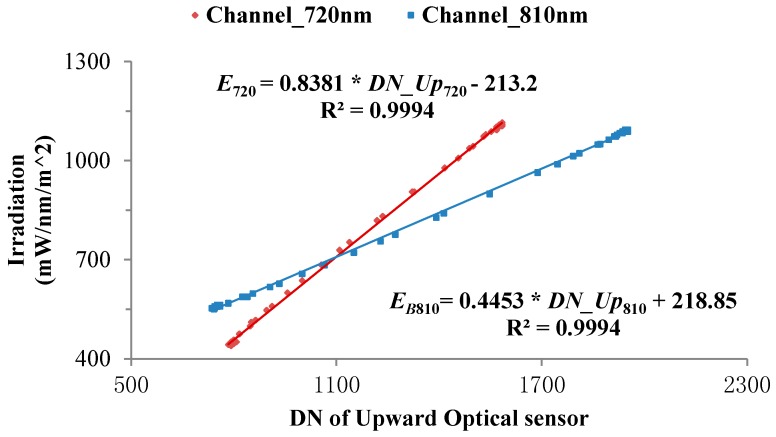
Calibration curve for irradiances received by the upward optical sensor.

By using the ASD spectrometer as the standard detector, irradiance from the upward optical sensor and radiance from the downward optical sensor are separately calibrated. Bare fibres of the ASD spectrometer, together with the standard cosine corrector, are fixed on the rotary platform at the same height as the upward optical sensor. After the ASD spectrometer has been set in irradiance mode, the angle of the platform is adjusted to enable sunlight to vertically impinge upon the upper surface of the upward optical sensor. Next, the platform is rotated slowly to simulate different solar altitudes and therefore form irradiance gradients. In addition, the test time needs to be as short as possible (usually 12:00 to 12:30) so as to ensure the stability of solar radiation intensity, as shown in Figure 10. The rotation angles of the platform, irradiances received by the ASD spectrometer at 720 and 810 nm, and output values of the upward optical sensor are separately recorded, so as to establish the calibration equation of the upward optical sensor, as shown in Equation (8).

(8) $$Er=Gain\_Up_{r}\times DN\_Up_{r}+Bias\_Up_{r}$$

where, *Gain_Up_r_* and *Bias_Up_r_* refer to the gain and drifting of the upward optical sensor, respectively. The calibration curve is shown in Figure 11.

A bare fibre of the ASD spectrometer and the downward optical sensor are separately fixed to the support. Then the height of the support is adjusted to make the downward optical sensor have the same observation field of view as the ASD. Six standard reflectance grayscale boards (SRT-10, SRT-20, SRT-40, SRT-60, SRT-75, and SRT-99) in the SRT series for calibration produced by Labsphere, (North Sutton, NH, USA) are selected as observed objects.

**Figure 12:**
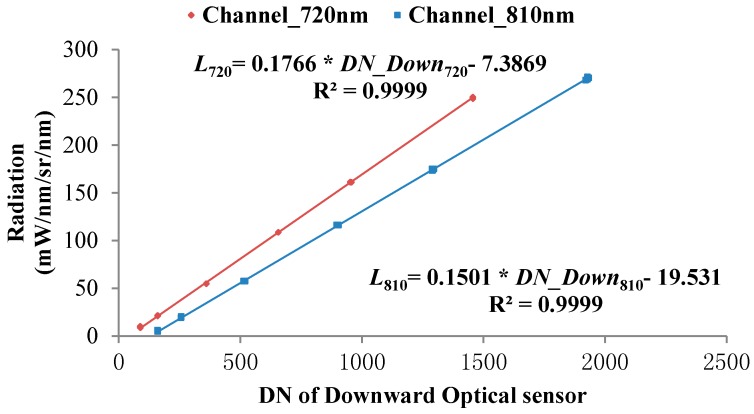
Calibration curve for radiances received by the downward optical sensor.

In addition, the ASD spectrometer is set to radiance measurement mode. Then, the radiances of the standard reflectance grayscale boards are measured vertically by using the downward optical sensor and the ASD spectrometer synchronously. Afterwards, the radiances obtained by the ASD spectrometer at 720 and 810 nm and the output values of the downward optical sensor are recorded, respectively to build the calibration equation of the downward optical sensor based on the ASD spectrometer, as shown in Equation (9):

(9) $$Lr=Gain\_Down_{r}\times DN\_Down_{r}+Bias\_Down_{r}$$

where *Gain_Down_r_* and *Bias_Down_r_* represent the gain and drifting of the downward optical sensor, respectively: the calibration curve is shown in Figure 12.

The calibrated upward and the downward optical sensors are assembled on the multispectral sensor. The target reflectance can be converted through use of Equation (3).

## 4. Field Experiment and Result Analysis

### 4.1. Experiment Design

The experiment was carried out at the Baipu test site of the National Engineering Research Centre for Information Technology in Agriculture in Nantong city, Jiangsu Province, China (120°46′ E, 32°16′ N), and yellow brown soil was used. The test varieties of wheat were Xumai 30 (XM) and Ningmai 13 (NM) and were planted on 30 October 2014 and harvested on 23 May 2015. The cumulative temperature and precipitation during the growth stage were 2146 °C and 412 mm, respectively. Five levels of nitrogen were applied, including N0 = 0 kg ha^−1^, N1 = 75 kg ha^−1^, N2 = 150 kg ha^−1^, N3 = 225 kg ha^−1^ and N4 = 300 kg ha^−1^. Each level was repeated three times, and therefore the experiments were conducted in 30 test fields, each of which covered an area of 5 m × 6 m. In addition, the line spacing and seeding depth of the wheat were 25 cm and 3 cm, respectively. The dressing ratio was 1:1. Phosphate fertilisers and potash fertilisers to masses of 0.36 and 0.39 kg were separately used in each test field. The test varieties of rice, that is Wuyunjing 24 (WY) and Y Liangyou 1 (LY), were planted on 13 June 2015 and harvested on 20 October of the same year. The cumulative temperature and precipitation during the growth stage were 3208 °C and 549 mm, respectively. Five levels of nitrogen were applied, including N0 = 0 kg ha^−1^, N1 = 150 kg ha^−1^, N2 = 225 kg ha^−1^, N3 = 300 kg ha^−1^, and N4 = 375 kg ha^−1^. Three duplications were set for each level, and therefore the experiments were conducted in 30 test fields, each of which was measured 6 m × 7 m. In addition, the rice was transplanted at intervals of 15 cm × 30 cm. The following fertilisers were used in the ratio of fed-base fertiliser:tillering fertiliser:spikelet-promoting fertiliser:spikelet-developing fertiliser of 4:1:3:2 allied with the aforementioned doses of phosphorus potassium fertilisers.

### 4.2. Data Collection

#### 4.2.1. Collection of Spectral Data

**Figure 13:**
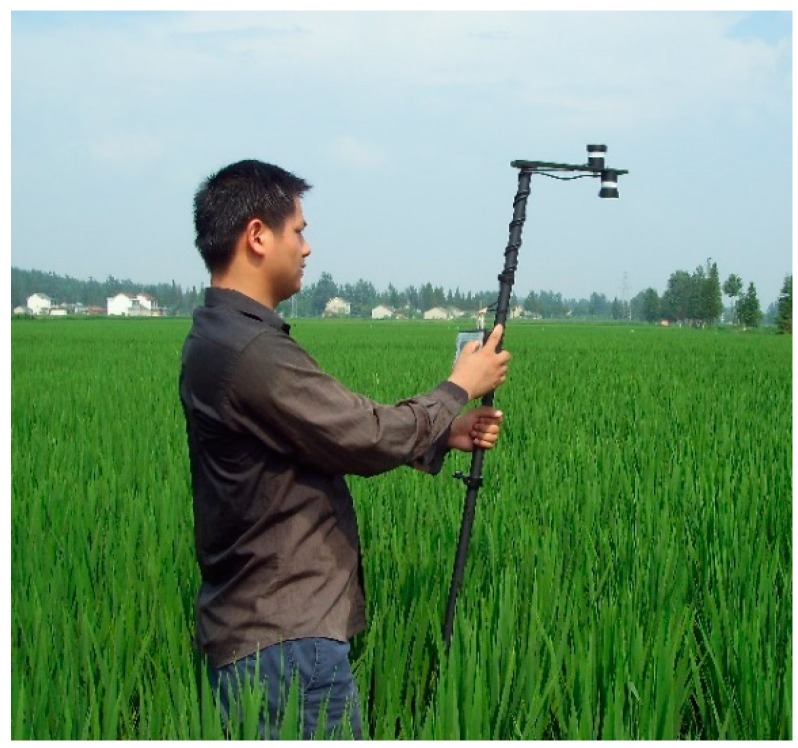
Collection of spectral data by using CGMD.

Canopy spectra were acquired by applying the calibrated CGMD apparatus and the ASD spectrometer. The downward optical sensor of the CGMD apparatus was placed 1 to 1.2 m above the canopy to collect data, as shown in Figure 13.

Six points in each test field were measured once and then the average was calculated and recorded. The fibre-optic probe of the ASD spectrometer was set at a distance of 1 m from the canopies, where the ASD spectrometer collected and recorded data synchronously with the CGMD apparatus. Canopy spectra were determined from 10:00 to 14:00 and in the absence of cloud and strong wind. Each experiment was undertaken to detect the crucial growth stages (elongation, booting, heading, and filling).

#### 4.2.2. Determination of Agronomic Parameters

At the same time as the spectral measurement, crops in the field were sampled using a destructive sampling method. In the sampling of the wheat, the numbers of wheat plants in 1 m long single lines were counted and recorded. In each test field, 10 to 15 plants were taken as samples to conduct indoor sampling. In addition, the total leaf areas *S* were measured by using the LAI3000 leaf area meter and the LAI of the whole test field was calculated by applying Equation (10). The samples were subjected to green-killing treatment for 30 min at 120 °C, and then dried at 80 °C. Next, the LDW *M* was measured and then LDW of the test field was calculated by employing Equation (11). Through further pulverisation, the LNC of the samples was determined by using the Kjeldahl method. Finally, according to Equation (12), the LNA was calculated:

(10) LAI=(S/n)×N/d

(11) LDW=(M/n)×N/d

(12) LNA=LNC×LDW

where, *d* refers to the line spacing of the wheat plants.

As for the rice, three rice plants were taken from each test field, followed by indoor sampling. The total leaf area *S* was determined by using an LAI3000 leaf area meter and the LAI of the whole test field was calculated by applying Equation (13). The samples were heated at 120 °C for 30 min (green-killing treatment) and then dried at 80 °C. Next, the LDW *M* was determined and then LDW of the test field was calculated by applying Equation (14). Through further pulverisation, the LNC of the samples was measured by using the Kjeldahl method. Finally, LNA was computed according to Equation (12):

(13) LAI=S/3/(h×l)

(14) LDW=M/3/(h×l)

where, *h* and *l* represent the line width and row spacing of the rice plants, respectively.

### 4.3. Results Analysis

#### 4.3.1. Performance of Spectral Information Monitoring with the CGMD Apparatus

**Figure 14:**
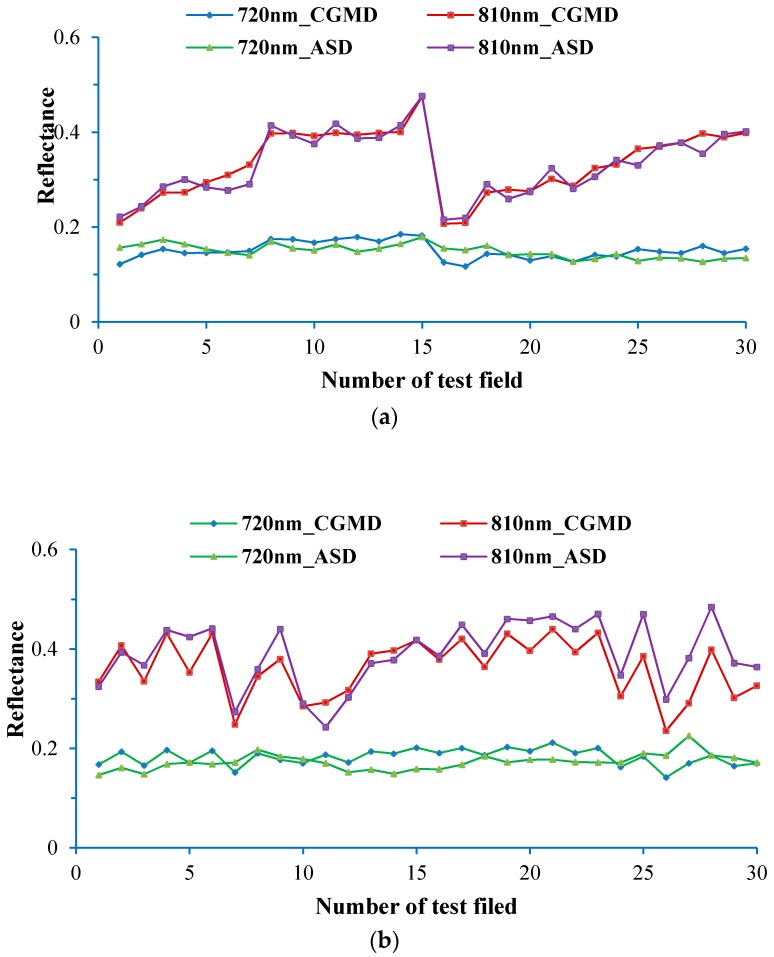
(**a**) Wheat; (**b**) Rice; Measurement of crop-canopy reflectance.

The reflectance of rice and wheat canopies measured by separately using the CGMD apparatus and the ASD spectrometer is displayed in Figure 14a,b.

The measured result arising from use of the CGMD apparatus was close to that found by applying the ASD spectrometer, both of which changed in a unified trend. Through statistical analysis, compared with the measured result from the ASD spectrometer, the average errors of canopy spectral reflectance of wheat at 720 and 810 nm collected using the CGMD apparatus were 2.03% and 2.01%, while the RMSEs were 1.84% and 1.83%, respectively. In addition, the average errors in the reflectance spectra of rice canopies at 720 and 810 nm measured applying the CGMD apparatus were 2.18% and 3.64%, while the RMSEs were 2.62% and 4.47%, respectively. It can be seen from the figures that the waveband at 720 nm showed a lower sensitivity to changes in the reflectance spectra of rice and wheat canopies under different treatments. So, the reflectance at this waveband exhibited an insignificant change, so the results at the waveband were taken as a reference for future comparisons and benchmarking. In comparison, the waveband at 810 nm was more sensitive to changes in the reflectance spectra of rice and wheat canopies under different treatments. So 810 nm was regarded as a sensitive band. The monitoring data were analysed by constructing the NDVI and the vegetation indices measured using the CGMD apparatus and the ASD spectrometer were selected for least squares fitting using a polynomial with degree of freedom, as shown in Figure 14. In addition, the NDVI is given by Equation (15):

(15) $$NDVI=(\rho_{810}-\rho_{720})/(\rho_{810}+\rho_{720})$$

**Figure 15:**
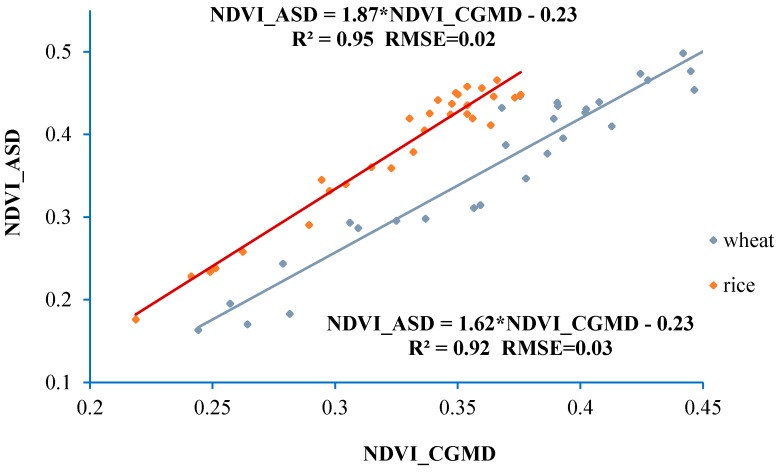
Fitting curve of NDVI measured using the CGMD apparatus and the ASD spectrometer.

Figure 15 shows that NDVI measured by using the CGMD apparatus has a good linear correlation with that obtained using the ASD spectrometer. Linear determination coefficients (*R*^2^) of the reflectance spectra of rice and wheat canopies were 0.95 and 0.92, while the RMSEs of their reflectance spectra were 0.02 and 0.03, respectively. The data indicate that spectral information monitored using the CGMD apparatus shows a high accuracy and has a strong correlation with measured results of the ASD spectrometer.

#### 4.3.2. Crop-Growth Information from Monitoring with the CGMD Apparatus

**Figure 16:**
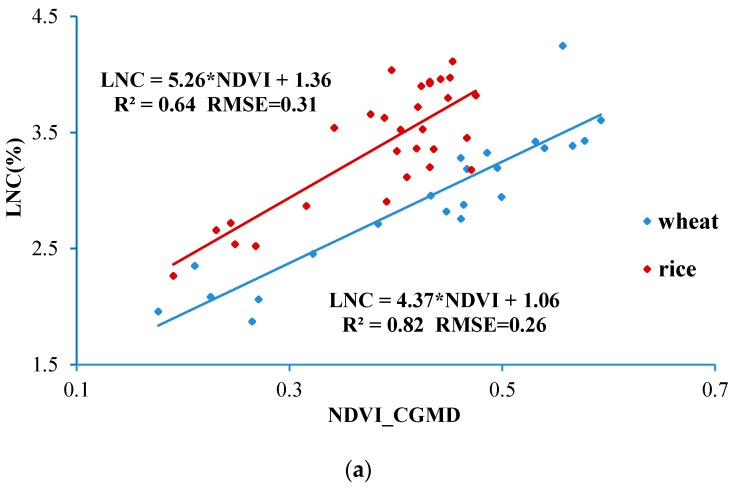

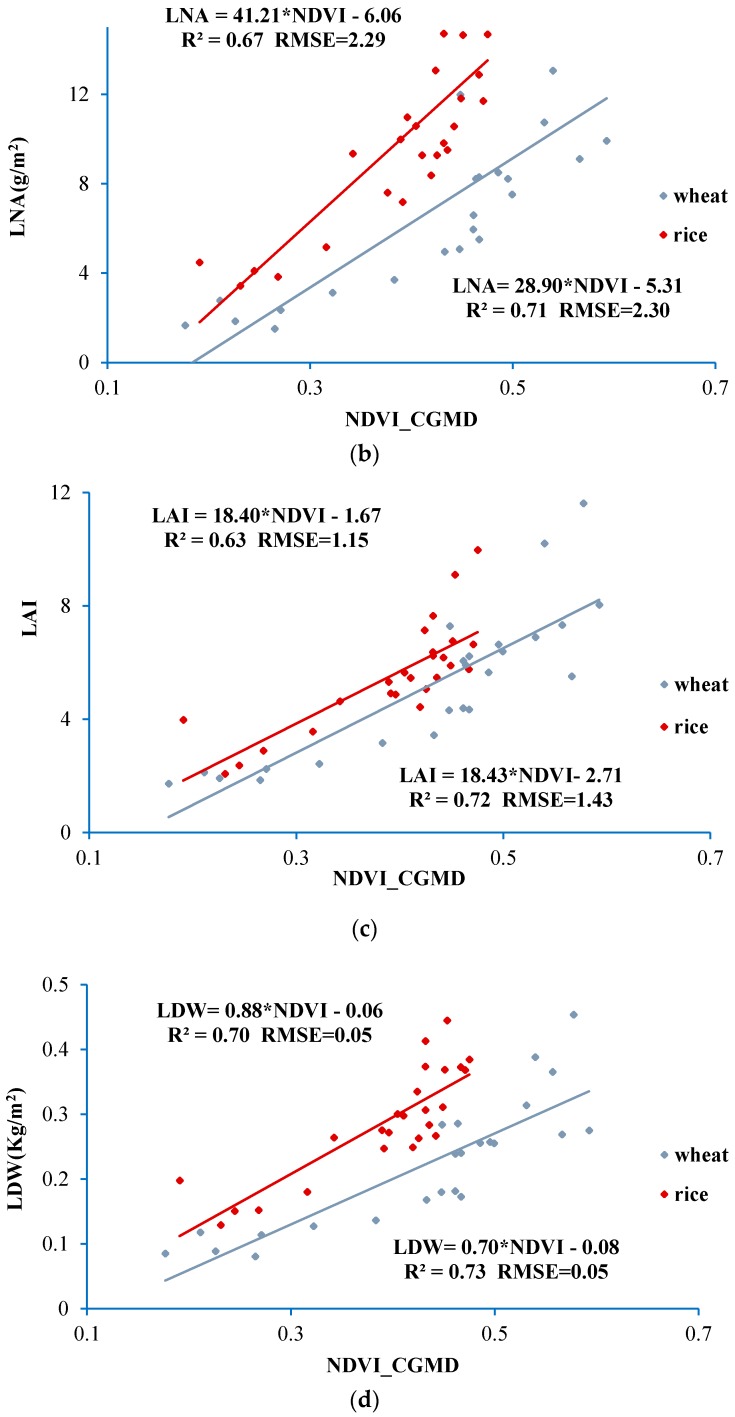
(**a**) Fitting curve of LNC-NDVI; (**b**) Fitting curve of LNA-NDVI; (**c**) Fitting curve of LAI-NDVI; (**d**) Fitting curve of LDA-NDVI; Spectral monitoring models for crop growth.

Linear least squares fitting was conducted for the NADI data measured by the CGMD apparatus and growth indices of rice and wheat including LNC, LNA, LAI, and LDW. The results are shown in Figure 16. Determination coefficients and RMSEs of the models were used to evaluate the prediction performance of the model.

It can be seen from Figure 16 that the NDVI value measured by using the CGMD apparatus exhibited a linear relationship with growth indices of rice and wheat. For the monitoring models for LNC, LNA, LAI, and LDW of rice based on linear fitting of NDVI data, the determination coefficients (*R*^2^) were 0.64, 0.67, 0.63 and 0.70, and RMSEs were 0.31, 2.29, 1.15 and 0.05 respectively. In addition, the determination coefficients (*R*^2^) of the models on the basis of the linear fitting of the NDVI for monitoring LNC, LNA, LAI, and LDW of wheat were 0.82, 0.71, 0.72 and 0.70 respectively and RMSEs were 0.26, 2.30, 1.43 and 0.05, respectively. The models exhibit a high accuracy and low error as well as a strong prediction capacity. By downloading the aforementioned models to the processor of the CGMD apparatus, it can predict growth conditions of both rice and wheat. Furthermore, the determination coefficients of spectral monitoring models for LNC, LNA, LAI and LDW of wheat were all larger than those for rice. This is probably because the water in the rice field had a significant influence on the measurement of canopy reflectance spectra before the closure stage of the rice, which caused a larger RMSE in the models for rice-growth spectral monitoring.

#### 4.3.3. Verification of the Models for Crop-Growth Spectral Monitoring

To verify the accuracy of the models for crop-growth spectral monitoring, three fields were randomly selected from the test site: the result is shown in Table 2.

It can be seen from the data analysis in Table 2 that real-time, on-line monitoring of growth information for rice and wheat can be achieved using the CGMD apparatus. In addition, the average measurement errors of NDVI, LNC, LNA, LAI and LDW were 5.96%, 10.35%, 8.23%, 4.68% and 5.24% respectively.

## 5. Discussion

Non-destructive monitoring technologies based on feature identification through reflectance spectra have been widely used in crop-growth monitoring, which promotes research into, and application of, crop-growth monitors based on spectral characteristics [36,37,38]. Some research institutes in China, and abroad, have studied the correlation between canopy reflectance spectra and nutrient levels in crops using commercial ground-based object spectrometers such as the hyper-spectrometer ASD-FieldSpec3 and multispectral Cropscan spectrometer [39,40,41]. These have broad wave bands and offer high-resolution data. For example, the measurement wave bands of ASD-FieldSpec3 spectrometers are between 350 and 2500 nm and the spectral resolution is 2 nm, giving these spectrometers a high measurement accuracy; however, the apparatus is expansive and has a complex structure and can be cumbersome to operate: calling for calibration with white boards before each measurement. These disadvantages limit the field popularisation and application of the apparatus. To reduce the application cost and improve ease of use, some research institutes have developed portable spectrometers with fewer wave bands. These include the two-band Greenseeker spectrometer (working at 656 and 774 nm) developed by Trimble Navigation, the two-band RapidScan CS-45 spectrometer (730 and 780 nm), and triple-band CropCircle ACS-470 spectrometer (660, 730 and 780 nm) developed by Holland Scientific [42,43,44,45]. The application of these spectrometers provides powerful technical support for information acquisition in the precision management of crops. Despite this, these pieces of apparatus have single functions, can only output canopy reflectance or spectral vegetation indices, and are mainly used in scientific research. Due to the lack of crop-growth information, they cannot be directly used in agricultural production. Besides, they are expensive and therefore fail to satisfy the requirements of precision agriculture for the low-cost acquisition of field information. Konica Minolta designed a two-band spectrometer SPAD-502 (working at 650 and 940 nm) which is able to output leaf chlorophyll content [46,47], however, it can only be applied to leaves and cannot measure the chlorophyll content of a crop community, so it cannot provide effective information for the precision management of crops. The CGMD apparatus works at two wave bands (720 and 810 nm) sensitive to nitrogen in rice and wheat and uses sensors with optical filters for beam-splitting. In their circuit design, integrators were used for the automatic adjustment of system gain, so it is convenient to use CGMD apparatus in the field. It can be seen from the results of a comparative test with the commercial hyper-spectrometer ASD-FieldSpec3 that the NDVI value of the canopy measured using the CGMD apparatus showed a favourable linear relationship and little error. Due to the elimination of real-time correction, the apparatus can be used in a convenient way at low cost and has more popularisation and utilisation prospects in the measurement of spectral vegetation indices of plant canopies. The CGMD apparatus incorporates established monitoring models for LNC, LAN, LAI, and LDW on its microcontroller chip, so it can be directly used in crop production management. Two different types of rice and wheat were used in the experiment. In future research, verification tests are needed at different ecological sites for different varieties of rice and wheat to improve the stability and universality of the monitoring models of the CGMD apparatus.

## 6. Conclusions

(1) A portable, low-cost CGMD apparatus was developed. The apparatus shows good cosine correction effect with an RMSE of 0.03 when the solar altitude is larger than 20°. The diameter and hole depth of the aperture of the sensors are designed to be 12.8 mm and 26 mm, respectively. Using an optical filter as the beam-splitting system, the sensors are of a simple structure and offer strong reliability. An integral-type processing circuit for weak optoelectronic signals is designed, which changes the gain of the system and guarantees the high resolution of the apparatus by automatically adjusting the integration period based on the irradiance of the ambient light.

(2) Experiments showed that NDVI data measured by using the CGMD apparatus exhibited a good linear correlation with those acquired using the ASD spectrometer. The linear determination coefficient (*R*^2^) of reflectance spectra of rice canopies was 0.95 while that of wheat canopies was 0.92. The spectral information monitored using the CGMD apparatus showed a high accuracy. Compared with the results measured using the ASD spectrometer, the average error of NDVI of rice and wheat measured using the CGMD apparatus was 5.96%.

(3) Field experiments showed that NDVI value measured by using the CGMD apparatus exhibited a linear relationship with growth indices of both rice and wheat. For the monitoring models for LNC, LNA, LAI, and LDW of rice based on linear fitting of NDVI, the determination coefficients (*R*^2^) were 0.64, 0.67, 0.63 and 0.70, respectively. In addition, the determination coefficients (*R*^2^) of the models for monitoring LNC, LNA, LAI, and LDW of wheat on the basis of the linear fitting of NDVI data were 0.82, 0.71, 0.72 and 0.70, respectively. The results showed that the CGMD apparatus showed a high accuracy in monitoring crop-growth information, and the average measurement errors of LNC, LNA, LAI, and LDW for rice and wheat were 10.35%, 8.23%, 4.68% and 5.24% respectively.

## Figures and Tables

**Table 1:** Relationship between zenith angle range and RMSE.

Zenith Angle	RMSE	Zenith Angle	RMSE	Zenith Angle	RMSE
0°~10°	0.0026	0°~40°	0.0148	0°~70°	0.03
0°~20°	0.014	0°~50°	0.0141	0°~80°	0.05
0°~30°	0.0165	0°~60°	0.0235	0°~90°	0.09

**Table 2:** Verification data of the CGMD apparatus. PV indicates predicted value; MV indicates measured value; RE indicates Relative error.

Cultivars	NDVI			LNC (%)			LNA (g/m^2^)			LAI			LDW (Kg/m^2^)		
	PV	MV	RE (%)	PV	MV	RE (%)	PV	MV	RE (%)	PV	MV	RE (%)	PV	MV	RE (%)
XM	0.51	0.51	0.00	3.29	3.45	4.62	9.43	10.21	8.20	6.69	6.71	0.19	0.28	0.27	3.11
NM	0.46	0.44	4.35	2.99	3.40	13.76	7.41	8.67	16.97	5.40	5.23	3.17	0.23	0.22	5.89
XM	0.46	0.42	8.70	2.90	3.23	11.42	6.83	7.28	6.62	5.03	4.96	1.55	0.21	0.21	4.38
WY	0.47	0.44	6.38	3.67	4.01	9.21	12.07	12.25	1.45	6.43	6.42	0.16	0.33	0.32	2.47
LY	0.31	0.28	9.68	2.83	2.60	8.09	5.48	5.17	5.67	3.48	3.11	10.61	0.19	0.17	9.26
WY	0.45	0.42	6.67	3.57	4.10	14.98	11.25	12.43	10.48	6.06	6.81	12.40	0.31	0.33	6.35
Average error	5.96%			10.35%			8.23%			4.68%			5.24%		
